# Overexpressing *Phytochrome* *Interacting* *Factor 8* of *Myrothamnus flabellifolia* Enhanced Drought and Salt Tolerance in Arabidopsis

**DOI:** 10.3390/ijms23158155

**Published:** 2022-07-24

**Authors:** Zhuo Huang, Rong Tang, Xin Yi, Wenxin Xu, Peilei Zhu, Cai-Zhong Jiang

**Affiliations:** 1 College of Landscape Architecture, Sichuan Agricultural University, Wenjiang 611130, China; 2020210023@stu.sicau.edu.cn (R.T.); jerrysyee@gmail.com (X.Y.); xuwenxin@stu.sicau.edu.cn (W.X.); zhupeilei1993@163.com (P.Z.); 2 Department of Plant Sciences, University of California Davis, Davis, CA 95616, USA; caizhong.jiang@usda.gov; 3 Crops Pathology and Genetics Research Unit, United States Department of Agriculture, Agricultural Research Service, Davis, CA 95616, USA

**Keywords:** *Myrothamnus flabellifolia*, resurrection plant, drought tolerance, Arabidopsis, basic helix–loop–helix (bHLH), phytochrome interacting factor (PIF)

## Abstract

*Myrothamnus flabellifolia* is the only woody resurrection plant found in the world and can survive from long-term desiccation. Therefore, *M. flabellifolia* could be considered as a valuable resource for study of plant adaptation to abiotic stress. However, few genes related to its drought tolerance have been functionally characterized and the molecular mechanisms underlying the stress tolerance of *M. flabellifolia* are largely unknown. The phytochrome interacting factor (PIF) family is a group of basic helix–loop–helix (bHLH) transcription factors and functions as the core regulator in plant growth and development. However, less is known of its participation in abiotic stress response. In this study, we isolated and characterized a dehydration-inducible PIF gene *MfPIF8* from *M. flabellifolia*. Heterologous expression of *MfPIF8* in Arabidopsis enhanced tolerance to drought and salinity stresses at seedling and adult stages. It significantly increased primary root length and stomatal aperture (ration of length/width) under stress treatments and decreased water loss rate. Compared with WT, the transgenic lines overexpressing *MfPIF8* exhibited higher chlorophyll content and lower malondialdehyde accumulation. The abilities of osmotic adjustment and reactive oxygen species scavenging were also enhanced in *MfPIF8* transgenic lines. These results suggest that MfPIF8 may participate in the positive regulation of abiotic stress responses. Additional investigation of its mechanism is needed in the future.

## 1. Introduction

Abiotic stresses such as drought, salinity, and extreme temperature detrimentally impact plants’ growth, development, and yield [1,2]. In order to respond to diverse abiotic stresses and increase the probability of survival, plants have evolved sophisticated mechanisms to regulate their responses to stresses, which are controlled by complex regulating networks involving a wide range of genes [3]. The genes encoding transcription factors (TFs) are among the most pivotal regulators to strengthen the plant adverse resistance [4]. It has been proved that overexpression of a single transcription factor can significantly improve the stress tolerance of transgenic plants [5]. Consequently, there is great significance in exploring the TF genes related to stress tolerance for the genetic improvement of plants.

Phytochrome-interacting factors (PIFs) and PIF-like (PILs) proteins belong to the 15th subfamily of basic helix–loop–helix (bHLH) transcription factor family [6]. They can directly interact with the activated Pfr (far-red light absorption type) form of the phytochrome (Phy) (phy–Pfr). All members of the PIFs family have a conserved N-terminal active phytochrome B-binding (APB) domain, and some members also have an active phytochrome A-binding (APA) domain at the N-terminal [7]. The APB domain contains four highly conserved amino acid sequences (ELXXXXGQ), which is essential for binding phyB–Pfr [8]. APA domain can interact with phyA–Pfr, but this domain is not conservative [9]. Additionally, as bHLH transcription factors, all PIFs also have one highly conserved bHLH domain, comprising a basic region and an HLH region followed closely, which bind to specific DNA sequences and promote protein–protein interactions, respectively [10,11].

PIF3 was the first transcription factor reported to interact with both phyB and phyA [12]. So far, there are eight members of PIF family (*AtPIF1-PIF8*) found in Arabidopsis [6,7], six members (*OsPIL11-OsPIL16*) in *Oryza sativa*, and seven members in *Zea mays* (*ZmPIF1-ZmPIF7*) [13]. Previous studies indicated that PIF family is the core regulator in plant growth and development, including inhibiting seed germination and seedling photomorphogenesis, promoting hypocotyl negative gravitropism and shade avoidance responses, and accelerating leaf senescence [14,15,16,17,18,19,20,21,22,23,24].

Additionally, PIFs have also been reported to respond to abiotic stresses by activating or inhibiting the expression of downstream genes by interacting with other proteins or protein complexes, or regulating the hormone level [25]. PIFs mediate the adaptive response of plants to drought stress mainly by changing the abscisic acid (ABA) level in vivo to control the number of stomata and the degree of opening and closing, regulate the transpiration rate, and maintain the water balance, which can achieve the drought resistance. Multiple *ZmPIFs* in *Zea mays* were drought-induced, in which both *ZmPIF1* and *ZmPIF3* enhanced drought resistance by reducing transpiration and leaf water loss rates [26]. Overexpressing *ZmPIF3* of *Z. mays* and *MfPIF1* of *Myrothamnus flabellifolia* in *Oryza sativa* and Arabidopsis, respectively, increased ABA biosynthesis, reduced stomatal opening, transpiration rate, and damage to the cell membrane system and significantly improved drought tolerance of transgenic plants [27,28]. Similarly, overexpression of *AtPIF3* in Arabidopsis improved drought tolerance with reduced transpiration rate [29]. The *PtPIFs* of poplar are responsive to low temperatures, drought, and salt stress. The expression level of *PtPIF8a* was significantly up-regulated about four times after drought stress, suggesting its potential role in *Populus* tolerance to drought stress [30]. Similarly, the expression of *StPIF8* was significantly up-regulated about five times under drought stress [31]. However, little is known of the functions of PIF8 in abiotic stress responses.

*M. flabellifolia* is the only woody resurrection plant discovered so far and mainly grows in mountainous areas of Central Africa and South Africa. To survive in the extremely dry mountain environment, the unique fan leaves of *M. flabellifolius* can fold and roll up tightly when plant tissue dehydrates, which makes the plants quickly turn into a long-term desiccant state and rehydrate rapidly after contact with water [32,33]. Despite a number of physiological and biochemical subjects in *M. flabellifolia* having been studied, the molecular mechanisms of extreme tolerance to desiccation and the ability to revitalize still remain unknown. Ma et al. [34] analyzed the transcriptome of *M. flabellifolia* and found that 295 transcription factors (TFs) actively responded to dehydration induction, and 287 TFs responded to rehydration, among which bHLH, MYB, and WRKY are predominant TF families involved in dehydration and rehydration responses. In this study, we isolated and characterized a bHLH gene *MfPIF8* (unigene ID: comp39737_c1_seq4), which was up-regulated at early dehydration stage. Overexpression of *MfPIF8* in Arabidopsis was performed to investigate its roles in drought and salinity stresses.

## 2. Results

### 2.1. Isolation and Characterization of MfPIF8

Based on the sequence of an early dehydration responsive unigene *comp39737_c1_seq4*, the primers were designed to isolate and re-sequence this gene from the cDNA of *M. flabellifolia*. The resulting amplified fragment contains 1377 bp nucleotide and encodes a putative protein of 458 amino acid residues. The protein has a calculated isoelectric point of 8.37 and a predicted molecular mass of 49.60 kDa. A Blastx search against protein sequences of Arabidopsis genome (Araport11) indicated that it shows the highest sequence similarity (E value = 2 × 10^−79^) with AT4G00050 encoding PHYTOCHROME INTERACTING FACTOR 8 (PIF8) (also named UNFERTILIZED EMBRYO SAC 10, UNE10), therefore named *MfPIF8*.

**Figure 1:**
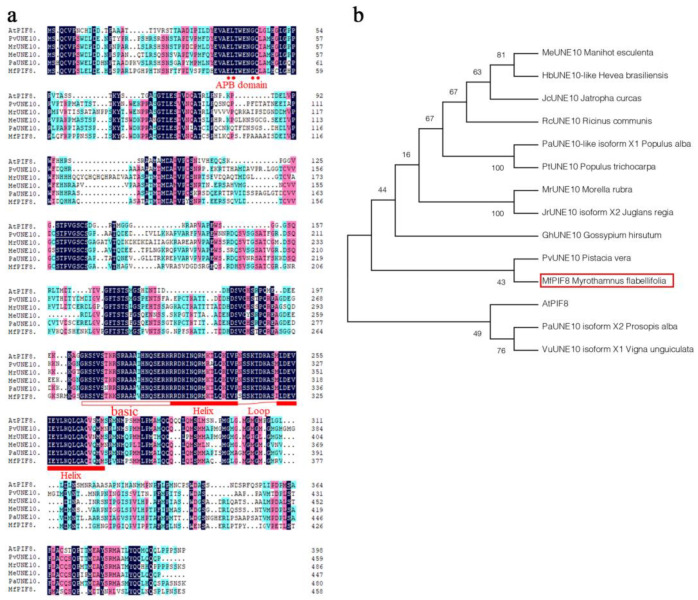
Multiple sequence alignment (**a**) and phylogenetic analysis (**b**) of MfPIF8 and several highly homologous bHLH proteins. (**a**) Identical and similar amino acids were highlighted. Conserved amino acid residues marked by red dots are active phytochrome B-binding (APB). The basic region was marked by the blank box, and the curve-linked red boxes indicate the conserved HLH domain. (**b**) Phylogenetic tree constructed using the neighbor-joining method. MfPIF8 is indicated by a hollow red box. The GenBank accession numbers and corresponding species for the sequences analyzed are listed in Table S1.

The multiple alignment of deduced amino acids of MfPIF8 and five highly homologous bHLH proteins showed that MfPIF8 has a putative APB domain at its N-terminus and a conserved basic region followed by an HLH domain. Comparing with homologous sequences, we found some amino acid variations of MfPIF8 in the bHLH domain, such as Arginine to Lysine (R to K) at position 281, Isoleucine to Valine at position 287, Asparagine to Serine at position 312, Methionine to Isoleucine at position 321, and Valine to Isoleucine at position 336 (Figure 1a). The subsequent phylogenetic tree revealed that MfPIF8 was most homologous to PvUNE10 of *Pistacia vera*. The two proteins were grouped to a separated clade (Figure 1b).

### 2.2. Overexpressing MfPIF8 Enhanced Tolerance to Drought and Salinity

To investigate the potential function of *MfPIF8* in response to abiotic stress, the genetic transformation of *MfPIF8* into Arabidopsis driven by CaMV 35S promoter was performed. T_1_ transgenic lines were obtained through kanamycin resistance screen, and the two T_3_ homozygous positive lines, line G and line R, were obtained by kanamycin screening and PCR and used for subsequent experiment. Quantitative real-time PCR showed that *MfPIF8* were expressed in Arabidopsis, in which line R showed a slightly higher expression level than that of line G (Figure S1).

**Figure 2:**
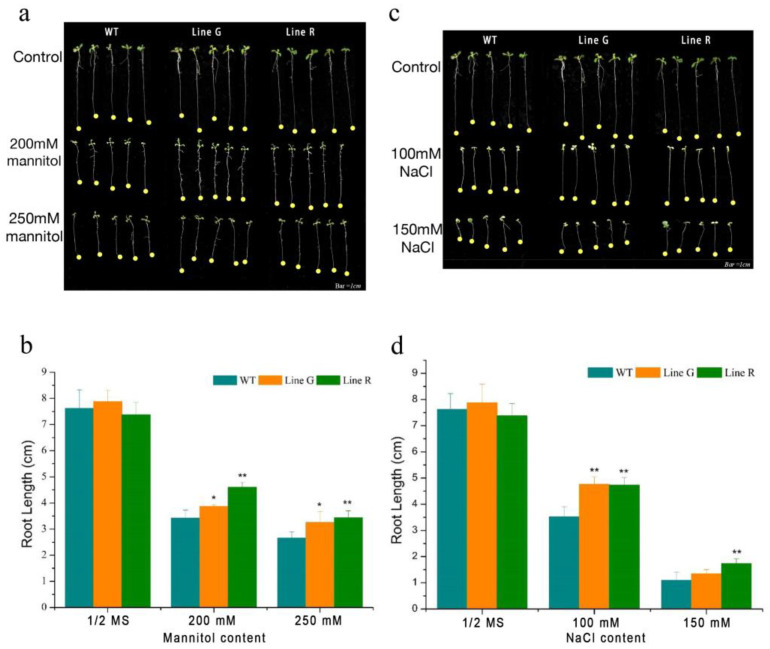
Phenotypic analyses of *MfPIF8* transgenic lines and WT under drought and salt treatments at the seedling stage. (**a**,**b**) Morphology and primary root length measurement of OE and WT seedlings grown on 1/2 MS medium with varying contents of mannitol for nine days. (**c**,**d**) Morphology and primary root length measurement of OE and WT seedlings grown on 1/2 MS medium with varying contents of NaCl for nine days. Data are presented as mean and SD values of three independent experiments. Asterisks indicate significant difference (* *p* < 0.05, ** *p* < 0.01, by independent sample *t*-test) compared with WT.

To further investigate the association between *MfPIF8* and abiotic stress tolerance, wild type (WT) and the overexpression (OE) lines were subjected to drought and salinity stress treatments and phenotypic analyses. At the seedling stage, there was no significant difference of primary root length between WT and OE plants under normal condition. Both mannitol and NaCl treatments inhibited root elongation in WT and OE lines. When treated by 200 mM and 250 mM mannitol, primary root length of line G (3.87 and 4.60 cm) and line R (3.25 and 3.43 cm) was significantly longer than the WT (3.43 and 2.66 cm) (Figure 2a,b). Under treatment of 100 mM NaCl, the primary root length of lines G and R was decreased to 4.76 and 4.73 cm, which were significantly longer than 3.52 cm of the WT. Under 150 mM NaCl treatment, the root length of line G was significantly inhibited to only 1.35 cm, which was slightly longer than WT (1.09 cm), while that of line R (1.73 cm) was significantly longer than that of WT (Figure 2c,d).

**Figure 3:**
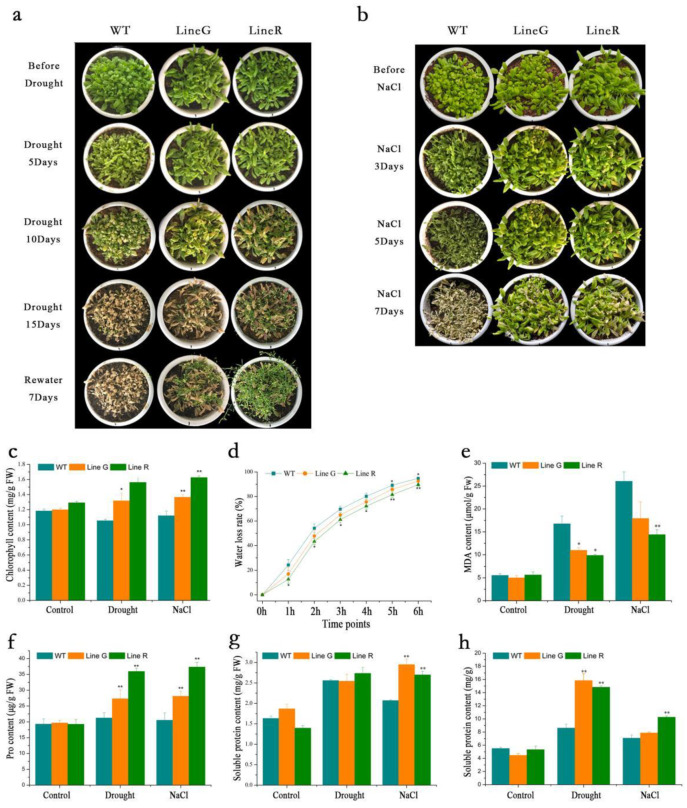
Evaluation of drought and salt tolerance and tolerance-related physiological and biochemical parameters at adult stage. (**a**,**b**) Indicates growth performance under drought and salinity treatments. (**c**–**h**) Shows measurements of tolerance-related physiological and biochemical parameters. Data are presented as mean and SD values of three independent experiments. Asterisks indicate significant difference (* *p* < 0.05, ** *p* < 0.01, by independent sample *t*-test) between WT and OE lines. In (**d**), the upper and lower asterisks indicate significant difference between WT and line G and between WT and line R, respectively.

At the adult stage, natural drought treatment by withholding watering and salt treatment by irrigation of 300 mM NaCl solution were applied to 4-week-old WT and OE plants. Before the treatments, the WT and OE lines were morphologically similar, except that the OE lines showed bigger shoots (Figure 3a,b). Five days after withholding watering (DAW), the leaves of WT and OE plants all became wilted (Figure 3a). At 10 DAW, the wilting degree was significantly increased, and at 15 DAW, the WT leaves were almost withered, while a certain amount of visible green parts could still be found on the two OE lines, especially the line R (Figure 3a). Seven days after rewatering, the two OE lines were significantly recovered and flowered, however, all WT plants died (Figure 3a). Similarly, the wilted phenotype under NaCl treatment was more serious in WT than those in OE lines. After seven days of salt treatment, almost all the WT plants died, whereas OE plants were still alive (Figure 3b). These results indicate that overexpression of *MfPIF8* in Arabidopsis enhanced drought and salt stresses.

We measured changes of chlorophyll content before and after treatments. Our results show that WT and OE lines had similar chlorophyll content before treatment. However, under drought and salt treatments, OE lines G and R exhibited significantly higher chlorophyll contents, which were 1.22–1.45-fold and 1.25–1.48-fold those of the WT, respectively (Figure 3c).

Water loss rate (WLR) is an indicator of water retention ability and closely related to drought tolerance. We found that lines G and R showed lower WLR at all time points investigated, and statistically significant differences were found at all time points between WT and line R. This result indicates that overexpression of MfPIF8 decreased WLR under drought (Figure 3d).

Malondialdehyde (MDA) accumulation is a common evaluation index of membrane oxidative damage. The MDA content of WT and OE lines were in a similar low degree under normal conditions. Both drought and salt induced MDA accumulation in WT and OE lines. However, WT accumulated significantly higher MDA content, which was more than 1.6-fold the average of the two OE lines (Figure 3e).

### 2.3. Effect of MfPIF8 Overexpression on Antioxidant Metabolism to Arabidopsis

**Figure 4:**
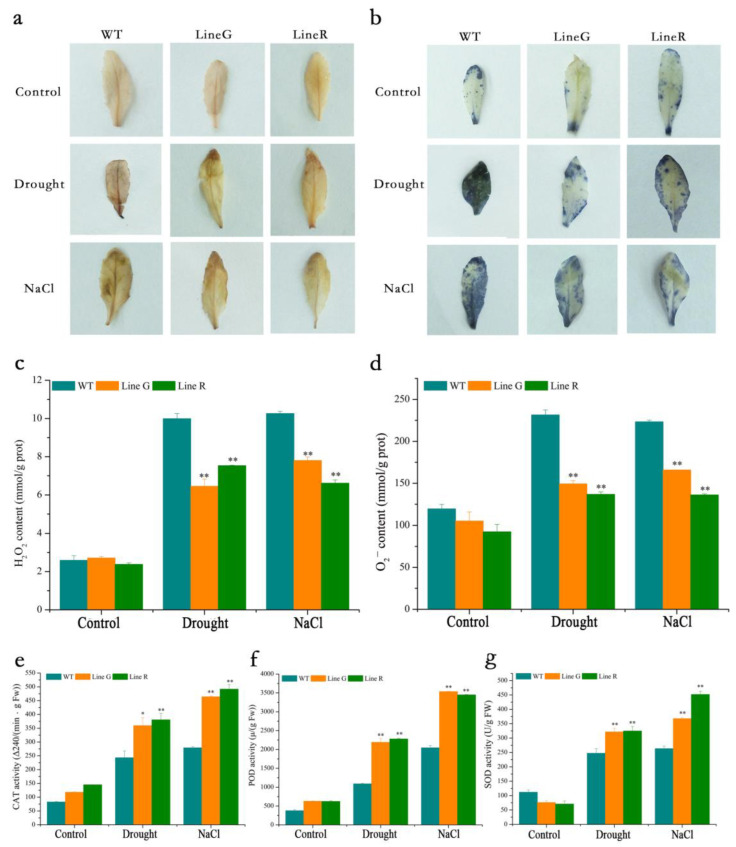
Analysis of reactive oxygen species (ROS) levels and antioxidant enzyme activities in *MfPIF8* transgenic and WT Arabidopsis. (**a**,**b**) Indicates histochemical staining by 3,3 -diaminobenzidine (DAB) and nitroblue tetrazolium (NBT), respectively. (**c**,**d**) Quantitative assays of H_2_O_2_ and O_2_^−^ contents in *MfPIF8* transgenic and WT Arabidopsis; (**e**–**g**) Indicates measurements of activities of antioxidant enzymes catalase (CAT), peroxidase (POD), and superoxide dismutase (SOD). Data are presented as mean and SD values of three independent experiments. Asterisks indicated significant difference (* *p* < 0.05, ** *p* < 0.01, by independent sample *t*-test) compared with WT.

Exposure to abiotic stress causes the accumulation of excessive reactive oxygen species (ROS), such as hydrogen peroxide (H_2_O_2_), superoxide anion radical (O_2_^−^), etc., in plant cells, and leads to an increase in lipid peroxidation along with cell oxidative damage. To determine the levels of ROS in cells under drought and salinity stresses, we used 3, 3′-diaminobenzidine (DAB) and nitroblue tetrazolium (NBT) histochemical staining. Under normal condition, the leaves of WT and OE lines were stained in very light brown, or little leaf area was stained in blue by DAB and NBT, respectively. After drought treatment, the leaves of WT were stained in deeper brown by DAB than those of the two OE lines, while less leaf area of OE lines was stained in lighter blue compared with WT (Figure 4a,b). Consistent with these results, quantitative assay showed that the two OE lines accumulated less contents of H_2_O_2_ and O_2_^−^ under both stresses (Figure 4c,d).

Antioxidant enzymes, such as peroxidase (POD), superoxide dismutase (SOD), and catalase (CAT), form an important plant defense system under environmental stress through ROS scavenging. Due to the low level of ROS accumulation in OE lines, we therefore further measured the activities of three antioxidant enzymes. Our results show that the activities of three enzymes were all increased in WT and OE lines along with the stress treatment. The two OE lines exhibited significantly higher activities of CAT, POD, and SOD under both drought and salt treatments, which were 1.52 and 1.71-fold, 2.05 and 1.71-fold, and 1.31 and 1.56-fold those of WT, respectively (Figure 4e–g). The above results indicate that introducing *MfPIF8* in the Arabidopsis increased activities of antioxidant enzymes and therefore decreased oxidative stress damage induced by drought and salt treatments.

### 2.4. MfPIF8 Overexpression Promoted Stomatal Closure Induced by Drought

**Figure 5:**
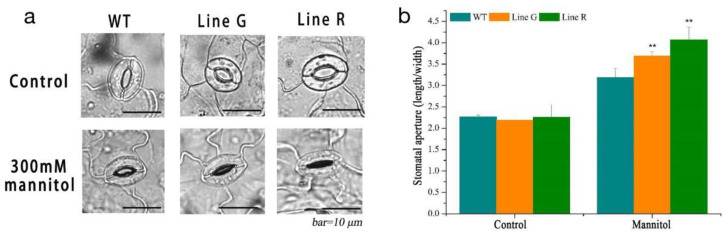
Stomatal movement of *MfPIF8* OE lines and WT in responding to 300 mM mannitol treatment. (**a**) Microscopy observation of stomata opening and closing degrees. (**b**) Changes in the stomatal aperture (ratio of stomata length/width). At least 60 stomata from 6 plants of each line were measured and three biological repeats were performed. Data are presented as mean and SD values of three independent experiments. Asterisks indicate significant difference (** *p* < 0.01, by independent sample *t*-test) compared with WT.

Stomatal movement is essential for plants to regulate transpiration in responding to unfavorable water conditions. We investigated stomatal movement under normal condition and treated by artificially simulated drought treatment (300 mM mannitol). No significant difference of stomata aperture was found between the WT and OE lines under normal condition. Induced by mannitol, the OE lines showed narrower stomata and higher stomata aperture (ratio of length/width) (Figure 5a,b), suggesting that stomata movement of the OE lines responded to mannitol treatment more quickly than the WT did.

## 3. Discussion

As a core factor of the phytochrome-mediated light signal pathway, PIF participates in diverse processes of plant growth and development and also mediates plant adaptation and defense responses to the changing environments. For example, transgenic plants simultaneously overexpressing *DREB1A* and *OsPIL1* can increase drought resistance [35]. Transgenic rice overexpressing maize *ZmPIF3* were more tolerant to dehydration and salt stresses than the controls [27]. The overexpression of *MfPIF1* of *M. flabellifolia* in *Arabidopsis* led to enhanced drought and salinity tolerance [28]. However, few studies revealed the functions of *PIF8* in abiotic stress tolerance. In the present study, we isolated and characterized *MfPIF8* from the woody resurrection plant *M. flabellifolia*, which was responsive to early dehydration [34]. *MfPIF8* contains a highly conserved bHLH domain and a typical APB domain (Figure 1a) and showed the highest homology with *PvUNE10* of *Pistacia vera* (Figure 1b). The overexpression of *MfPIF8* significantly improved the drought and salt tolerance of transgenic Arabidopsis plants both in seedling and adult stages (Figure 2 and Figure 3a,b), demonstrating that *MfPIF8* possibly acts as a positive regulator to the drought and salinity response.

Increasing plant tolerance to drought and salt stress under adversity involves multiple complex biological processes, such as changes in morphology and physiological and biochemical effects. Roots can perceive and pass environment stress signals to the rest of the plant, which initiate morphological, physiological, biochemical, and molecular responses [36]. Additionally, under adverse environmental conditions, enhancing the growth of primary roots would offer an advantage to the plants by expanding their domains of water supply [37]. Therefore, root length is one of the important indicators of drought tolerance [38]. In the seedling osmotic and salinity stress tests, the primary roots of two OE lines were significantly longer than the WT seedlings, which may contribute to better water absorption capacity (Figure 2a,c).

Approximately 90% of water loss (transpiration) occurs through stomata, and stomatal closure is the first step to reducing water loss by plants under drought stress [39,40]. Zhang et al. [41] found that under drought conditions, the OE lines showed improved water-use efficiency by reducing their stomatal conductance and transpiration rate, indicating that overexpression of *PtrWRKY75* enhanced the drought tolerance in poplars. In this study, the enhanced stomatal closure under mannitol treatment was found in *MfPIF8* OE plants (Figure 5). This result is consistent with the lower WLR under both drought and salinity stresses (Figure 3d). Thus, the enhanced primary root length and stomatal closure increased water uptake and retention capacity, and therefore enhanced drought and salt tolerance.

The amount of chlorophyll content greatly affects the level of photosynthesis efficiency under stress [42]. As stress-induced destruction of photosynthetic structure negatively affects plant photosynthesis. Existing studies showed that chlorophyll content in plants tends to decrease due to inhibition of chlorophyll synthesis and oxidative damage under stress [43,44]. Thus, chlorophyll content can be used as one of the indexes to measure drought tolerance of plants. Arabidopsis overexpressing *FtWRKY46* [45] and *MxWRKY55* [46] has higher chlorophyll content and increased salinity tolerance. In this study, we found that chlorophyll contents were significantly higher in OE lines than that in WT under drought and salt stresses (Figure 3c). This result suggests that overexpression of *MfPIF8* may promote generation or maintenance of chlorophyll from abiotic stress-induced oxidative damage in Arabidopsis.

A high degree of drought and salinity can cause similar damage to plants by imposing osmotic stress [47], while plants can response to osmotic stress through the accumulation of osmolytes [48]. The proline, soluble sugars, and soluble proteins are reported to act as osmoprotectants by maintaining the osmotic balance [49,50]. It was reported that the contents of proline and soluble sugars in *UGT3*-overexpressing plants were higher than that of in WT, and those of the *UGT3ko* mutant strains were less than that of WT in response to drought stress in rice [51]. In our study, the content of proline, soluble sugar, and soluble protein were generally increased in OE lines compared with WT. Under both drought and salt stresses, OE lines simultaneously accumulated significantly more proline. Moreover, two OE lines simultaneously accumulated significantly higher soluble protein content under salt stress and significantly higher soluble sugar content under drought stress, respectively. These results demonstrate that MfPIF8 directly or indirectly enhanced osmotic adjustment ability under drought and salinity stresses (Figure 3f–h). However, its roles in responding to drought and salt may be different.

Malondialdehyde (MDA), the product of lipid peroxidation, is a reflection of lipid peroxidation and is usually used to measure stress-induced damage [52]. Accumulation of MDA contents of salt-tolerant cultivars was lower than that of salt-sensitive cultivars under salt stress, which is strongly in agreement with the studies of Hassine [53] and Carrasco-Ríos [54] in peanut and maize, respectively. In this study, transgenic plants accumulated less MDA compared with WT, indicating that MfPIF8 could play a role in the decreasing lipid peroxidation (Figure 3e).

Oxidative damage to cellular components, caused by stress-induced excessive accumulation of ROS, such as O_2_^−^ and H_2_O_2_, leads to toxic effects on plant growth and development [55]. In response to such stress, plant cells have evolved a variety of antioxidant mechanisms, such as regulating the activities of antioxidant enzymes, such as CAT, POD, SOD [56]. It has been found that by increasing the activities of CAT, POD, and SOD, *MdbHLH130* can quickly remove reactive oxygen species in cells, maintain stable cell membrane structure and function, and improve drought tolerance of transgenic tobacco [57]. In the present study, we found that the activities of all three enzymes were significantly higher than that of WT (Figure 4e–g). Consequently, the ROS levels evaluated by histochemical staining and quantitative assay were also lower than that in WT under drought and salt treatments (Figure 4a–d). These results indicate that the ROS-scavenging capacity was significantly enhanced by introduction of *MfPIF8*. Taken together, our results in this study suggest that MfPIF8 could improve the drought and salt tolerance of Arabidopsis through increasing water uptake and retention capacity and osmotic adjustment ability and enhancing ROS-scavenging system. Therefore, *MfPIF8* has important application potential in the genetic improvement of plant stress tolerance. In the future, understanding the interacting proteins of MfPIF8 and the signal pathways it directly participates in deserves further study.

## 4. Materials and Methods

### 4.1. Plant Materials, Growing Conditions, and Stress Treatments

Wild type (WT) Arabidopsis ecotype *Columbia* was conserved by our lab. *M. flabellifolia* and was provided by the Department of Plant Sciences, University of California, Davis. The seeds of Arabidopsis were sterilized with 1:1 diluted bleach, washed with deionized water three times (1 min each time), and then planted in petri dishes with 1/2 MS (Murashige & Skoog) solid medium. After a 2-day vernalization (4 °C), the dishes were transferred into a incubator under conditions of 16 h light (24 °C)/8 h dark (22 °C) and 75% relative humidity. For the adult-stage experimental, the seedlings germinated in 1/2 MS solid medium were transplanted in pots containing an equal amount of mixture of soil and vermiculite (1:3 of *v*/*v*) and grown under condition of 24 °C (day)/22 °C (night), with 75% relative humidity and with a 16 h light/8 h dark photoperiod.

In seedling stage treatment, sterilized seeds were sown on 1/2 MS solid medium containing different concentrations of mannitol (0, 200 mM, 250 mM) and NaCl (0, 100 mM, 150 mM), respectively. After dark treatment at 4 °C for 2 days, the seeds were moved into a light incubator and cultivated vertically for 2 weeks, and the root lengths were measured. In adult-stage treatment, the germinated seedlings were transplanted in plastic pots and grown for 4 weeks under the same normal condition as described above. The plants in similar status were used for further analysis. For the drought treatment, the plants were fully irrigated and then the watering was stopped for several days. In salt treatment, the experimental groups were watered with a 300 mM NaCl solution twice at a 3-day interval and cultured for about 2 weeks. All plants were observed and photographed daily.

### 4.2. Cloning and Bioinformatic Analysis of MfPIF8

RNA of *M. flabellifolia* was extracted using the Plant Total RNA Isolation Kit (Lanbo Biotechnology Company, Chengdu, China). The cDNA was synthesized using the Reverse Transcriptase M-MLV (RNaseH-) kit (TaKaRa, Dalian, China). According to uniGene (comp39737_c1__seq4) sequence corresponding to *MfPIF8*, a primer pair was designed by SnapGene software and the sequences were: Forword: 5′-TCCCCCGGGATGAGCCAGTGCGTTCCCAG-3′ (*Sma* I site was underlined) and Reverse: 5′-GACTAGTTCAGCTCTCCGAGTTTGGAA3′ (*Spe* I site was underlined). The target PCR product amplified was recovered from the agarose gel (FastPure Gel DNA Extraction Mini Kit, Nanjing, China) and connected to pEasy-T1 simple vector (Transgen Biotechnology, Beijing, China). The obtained ligation product, pEasy-T1-MfPIF8, was immediately transferred into *Escherichia coli* strain DH5α. Finally, cloning of MfPIF8 was confirmed by sequencing (Chengdu Qingke Biotechnology Co., Ltd., Chengdu, China).

Open Reading Frame (ORF) prediction, molecular weight, and isoelectric point (pI) of protein were conducted and determined by SnapGene software. Prediction and analysis of the protein domains were performed by SMART (Available online: http://smart.embl-heidelberg.de/ (accessed on 20 July 2022)). Homologous proteins of MfPIF8 were searched by NCBI BLASTP (Available online: https://blast.ncbi.nlm.nih.gov/Blast.cgi (accessed on 20 July 2022)) against nr protein dataset. Then, multiple sequence alignment was performed by DNAMAN (version 5.2.2) (Lynnon Biosoft, Vaudreuil, QC, Canada), and the similarities and differences between conserved domains of homologous proteins were preliminarily analyzed. Finally, phylogenetic tree analysis was carried out by MEGA 6.0 software using the neighbor-joining method with the bootstrap test of 1000 replicates [58].

### 4.3. Vector Construction and Generation of Transgenic Lines

The pGSA-1403 plasmid and amplified target fragment were simultaneously digested by QuickCut restriction enzymes *Sma* I and *Spe* I (TaKaRa, Dalian, China). The overexpression vector pGSA1403-*MfPIF8* was constructed by ligated linearized vector and target fragment by using T4 DNA ligase (DNA Ligation Kit, Takara, Beijing, China). The resulted plasmid of pGSA1403-MfPIF8 was transferred into competent cells of *Agrobacterium tumefaciens* strain LBA4404. Arabidopsis was transfected by flower-dipping method [59]. Seeds of generation T_0_ were sown on 1/2 MS medium with kanamycin (Kan) (50 μg/mL), and repeated selfing and screening by Kan and PCR were performed. The homozygous positive T_3_ lines were obtained and used for further analysis.

### 4.4. Water Loss Rate

The 0.5 g rosette leaves of 4-week-old WT and transgenic plants were collected from at least 10 plants of each group to determine the natural water loss rate. The leaves were placed on filter paper under conditions of constant ambient humidity temperature (24 °C, 60% air humidity) to dehydrate naturally. Leaves were weighed at set time points (0, 1, 2, 3, 4, 5, and 6 h). The experiments were repeated three times.

### 4.5. Stomatal Aperture Analysis

To measure stomatal movement during drought, rosette leaves from at least 6 individuals of WT and OE line grown for 4 weeks and in similar growth status were soaked in MES buffer (50 mM KCl, 0.1 mM CaCl_2_, 10 mM MES, pH 6.15) to induce stomatal opening for 2.5 h and recorded as the control group. Then, the leaves were transferred to MES-KCl stomatal-induced open buffer supplemented with 300 mM mannitol and treated with light for 2 h and recorded as the experimental group. After the treatments, the stomata were observed and the width and length were measured by using an optical microscopy (DP80, Olympus, Tokyo, Japan). The length and width of at least 60 stomata (10 per plant) of each line were measured and the stomatal aperture (width to length ratio) was also calculated. All the experiments were repeated three times.

### 4.6. Measurements of Tolerance-Related Physiological and Biochemical Parameters

Chlorophyll content was extracted with 95% ethanol and measured according to previous reported [60]. To determine the osmotic stress substances, proline content was assayed by acid ninhydrin method [61], and the contents of soluble sugar and soluble protein were quantitatively determined with Plant Soluble Sugar Content Detection Kit (Nanjing Jiancheng, Nanjing, China) and Soluble Protein Total Protein Quantitative Determination Kit (Nanjing Jiancheng, Nanjing, China), respectively.

Leaf tissues were stained, respectively, using NBT and DAB dye to visualize the accumulation of reactive oxygen species (ROS) [62], followed by decolorization in 95% ethanol. A hydrogen peroxide assay kit and a superoxide anion assay kit (Nanjing Jiancheng, Nanjing, China) were used to measure the reactive oxygen species (ROS) level (H_2_O_2_ and O_2_^−^), respectively. The activities of antioxidant enzymes peroxidase (POD), superoxide dismutase (SOD), and catalase (CAT) were determined according to a previous report [63]. The content of malondialdehyde (MDA) was measured by thiobarbituric acid (TBA) method to judge the stability of the plasma membrane [64]. All the experiments were repeated three times.

### 4.7. Statistical Analyses

The data of this study were processed and analyzed using the Student’s *t*-test in SPSS (version 23.0) (IBM, Chicago, USA). The values were expressed as the mean ± standard deviation (SD) of three replicates, and significant differences were presented as * *p* < 0.05 and ** *p* < 0.01. 

## Data Availability

Not applicable.

## Supplementary Materials

The following supporting information can be downloaded at: https://www.mdpi.com/article/10.3390/ijms23158155/s1.
