# Supplementary material for: Overexpressing Phytochrome Interacting Factor 8 of Myrothamnus flabellifolia Enhanced Drought and Salt Tolerance in Arabidopsis
Source: Int J Mol Sci. 2022 Jul 24;23(15):8155. doi: 10.3390/ijms23158155 (PMC9331687; doi:10.3390/ijms23158155)
Supplement: Supplementary file 1 [file ijms-23-08155-s001.zip › ijms-1829231-supplementary.pdf]

Table S1 The GenBank accession numbers of the homologous sequences for MfPIF8 in different species.

ID	**Accession numbers**	**Species**
Pv UNE10	XP_031269671.1	*Pistacia vera*
Mr UNE10	KAB1225233.1	*Morella rubra;*
Me UNE10	XP_021614535.1	*Manihot esculenta*
Hb UNE10-like	XP_021677931.1	*Hevea brasiliensis*
Gh UNE10	XP_040961719.1	*Gossypium hirsutum*
Pa UNE10-like isoform X1	XP_034905753.1	*Populus alba*
Jc UNE10	XP_012083633.1	*Jatropha curcas*
Pa UNE10 isoform X2	XP_028804697.1	*Prosopis alba*
Rc UNE10	XP_015582839.1	*Ricinus communis*
Jr UNE10 isoform X2	XP_018853947.1	*Juglans regia*
Pt UNE10	XP_002320711.2	*Populus trichocarpa*
Vu UNE10 isoform X1	XP_027925895.1	*Vigna unguiculata*
AtPIF8	AT4G00050	*Arabidopsis*

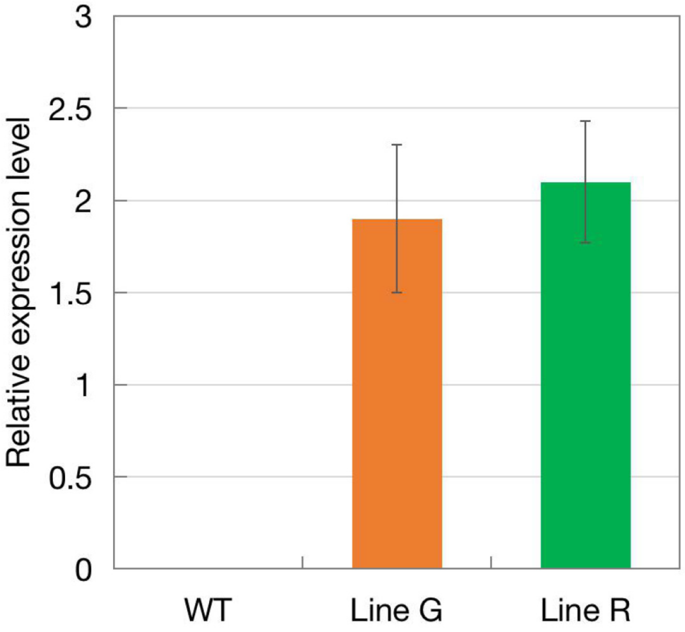

Figure S1 Analysis of expression level of *MfPIF8* by quantitative real-time PCR (qRT-PCR). Leaves were sampled from transgenic and WT plants grown to 4-week-old . RNA extraction, reverse transcription, qRT-PCR amplification, and expression level quantification were performed according to previous reported (Qiu et al. 2020)[28]. The *Actin2* was used as an internal reference. The gene-specific primers are: *Actin2F: 5′*-GGAAGGATCTGTACGGTAAC-3′ and *Actin2R: 5′*-TGTGAACGATTCCTGGACCT-3′ for *Actin2* and *MfPIF8F*: 5′-CAGCGGCACACTTGAGTCTA-3′ and *MfPIF8R*: 5′-TCATTGTGGCAGTAGCGGAG-3′ for *MfPIF8*, respectively.
